# Influence of Screen Time on Physical Activity and Lifestyle Factors in German School Children: Interim Results from the Hand-on-Heart-Study (“Hand aufs Herz”)

**DOI:** 10.3390/children12050576

**Published:** 2025-04-29

**Authors:** Jennifer Wieprecht, Delphina Gomes, Federico Morassutti Vitale, Simone Katrin Manai, Samar Shamas, Marcel Müller, Maren Baethmann, Anja Tengler, Roxana Riley, Guido Mandilaras, Nikolaus Alexander Haas, Meike Schrader

**Affiliations:** 1 Division of Pediatric Cardiology and Intensive Care, University Hospital, LMU Munich, D-81377 Munich, Germany; 2 Institute for Medical Information Processing Biometry and Epidemiology, University Hospital, LMU Munich, D-81377 Munich, Germany

**Keywords:** physical activity, screen time, BMI, adolescents, sports clubs, hand-on-heart-study (“Hand aufs Herz”)

## Abstract

**Background/Objectives**: Today, digital technologies are integral to children’s lives; their increasing use, however, may raise health concerns. This study aims to examine the effect of screen time on physical activity and lifestyle factors in German school children. **Methods**: As part of the prospective hand-on-heart-study (“Hand-aufs-Herz”), a comprehensive cardiovascular system check-up examination was conducted on 922 German schoolchildren. The pupils were asked for a self-report on their daily physical activities and club sports. The examinations on-site contained measurements of the pupils’ weight and height as well as their physical fitness, which was assessed by a stair-climbing test. **Results**: A large proportion of pupils had a screen time of more than 2 h daily, regardless of the day of the week (63–76%). In fact, pupils with a screen time ≥ 2 h were more likely to achieve poor grades in school (weekday ORs 3.23, 95% CI 1.76, 5.95; weekend ORs 3.28, 95% CI 1.53, 7.00) and not be members of a sports club (weekday ORs 2.35, 95% CI 1.68, 3.29; weekend ORs 2.13, 95% CI 1.44, 3.14). Pupils who did not meet both recommendations for physical activity and screen time walked <5000 steps daily (60%), had a high proportion of overweight/obesity (40%), were non-swimmers (38.5%), spent ≥7 h sitting (35.8%), and slept fewer hours than recommended (30%). It has also been shown that longer screen time has a negative impact on the lifestyle of children and young people. **Conclusions**: Our results show that excessive screen time in children is linked to higher weight and an unhealthy lifestyle, increasing long-term cardiovascular risks. Public health initiatives aimed at reducing screen time, promoting physical activity, and encouraging healthier habits are essential to improve children’s overall health and prevent future chronic diseases.

## 1. Introduction

Cardiovascular diseases (CVDs) remain the number one cause of death in industrialized countries and account for about 11% of health expenses in Europe [1]. Cardiovascular diseases not only affect adults but can also originate in childhood, even if they do not manifest themselves at that time [2,3].

The risk factors for future CVDs can exist during childhood [4] but are modifiable [5]. The importance of physical activity (PA) in preventing CVDs has been widely recognized and regular exercise helps improve cardiovascular health by lowering blood pressure, reducing cholesterol levels, improving blood circulation, and preventing obesity, all of which are key risk factors for heart disease [6,7]. It has already been shown that overweight and obese adolescents have significantly lower cardiorespiratory fitness than their normal-weight peers [8]. In fact, lower physical fitness is a proven independent risk factor for all-cause mortality, regardless of weight status [9].

Despite the known benefit of physical activity [10], 81% of adolescents aged 11–17 years worldwide are physically inactive [11]. In Germany, most children and adolescents are inactive [12], with studies revealing that only 22% of girls and 29% of boys aged 3–17 meet the World Health Organization’s (WHO) recommendation of being active for at least 60 min a day. The proportion of children who fulfil the recommendations decreases continuously with age [3,4]. The rising levels of inactivity are concerning, particularly in relation to the growing amount of screen time, which has become a major factor in promoting sedentary behavior among young people [1]. Children and youth today are growing up in a world where both traditional and modern digital media are an integral part of everyday life [13]. Traditional media, such as television, coexist with newer formats like apps, video games, YouTube, and vlogs. Televisions, smartphones, computers, laptops, and internet access are now standard in almost every household [14,15].

Over the past decade, young people have increasingly spent more time using digital media [14]. A large study from 22 countries has found that more than 1 in 10 adolescents (11%) have signs of problematic social media behavior, struggling to control their use and experiencing negative consequences [16]. In 2022, a survey conducted in Germany with 1219 children aged 6 to 13 years found that 81% of 12- to 13-year-olds already owned their own smartphone. Television remained one of their favorite leisure activities, with 92% of children watching television regularly, and 67% even watched it every day [15]. Just two years later, almost every child between the ages of 12 and 19 years owned a smartphone [17]. A more recent German survey showed that screen time increases with age: 10–12-year-olds spent an average of 87 min per day online, 13- to 15-year-olds spent 140 min, and 16- to 18-year-olds spent 166 min [18]. These figures exceed the recommended screen time limits, which suggest a maximum of 45 to 60 min for children between the ages of 9 and 12 and 120 min for adolescents aged 12 to 16 years [19]. Not only might media consumption have an impact on children’s physical activity and fitness; participation in club sports also plays a decisive role. Young people who regularly took part in organized sport activities, whether in clubs or at school, showed a higher overall level of activity and better physical fitness than their peers who did not participate [8].

In addition to the alarming trends in screen time and physical activity among children and adolescents, studies also show worrying results concerning sleeping habits. A study of German schoolchildren aged 9 to 18 found that only half of them were getting the recommended amount of sleep. If the recommendations for screen time and exercise are also taken into account, only 9.7% of children met all three guidelines [20].

To summarize, there is a worrying trend about sleeping habits, lack of exercise, and increasing screen time. Obesity and a lack of physical activity are among the main risk factors for cardiovascular disease. This raises the question of the extent to which increased media consumption not only reduces daily exercise but also contributes to weight gain and has a negative impact on other lifestyle habits. In the present study, we investigated the influence of screen time on physical fitness and lifestyle by combining the results of a self-report on daily physical activities with a standardized fitness test.

## 2. Materials and Methods

### 2.1. Study Population

In this ongoing study, a total of 922 pupils at a secondary school in the rural Munich area, including siblings, were enrolled between April and July 2024. The consent of both parents was required for participation in our study. For the current analysis, young adults (aged 18 to 20 years) were excluded, and the data of 883 children and adolescents aged between 8 and 18 years were analyzed.

### 2.2. Procedure

The study presented here bases itself on data collected with the project hand-on-heart (“Hand-aufs-Herz”, German Clinical trial register DRKS00033999), a prevention project of the Department of Pediatric Cardiology of the University Hospital Munich [21]. Ethical approval to perform this study was granted on 15 April 2024 with the project number (24-0147) by the Ethics Committee of the Ludwig-Maximilians-Universität.

At the beginning, the project was presented to the parents in a letter and with information flyers handed out to school management and supervising teachers. This was followed by a parents’ evening at which the parents were informed again in detail. The project was presented to all pupils on a class-by-class basis. Registration was carried out via a written form; the signature of both parents was required for participation. The tests were carried out by our study team in the school. All participants presented themselves once, and parents were informed of the dates in advance by email. The examinations lasted 45 to 60 min per participant, of which 15 to 20 min were needed to answer the questionnaire on a tablet.

The study team consisted of three doctors and a total of ten students from the healthcare sector. Two doctors and three to four students were present per day. The doctors are members of the Pediatric Cardiology Department of the University Hospital Munich and are experienced in the diagnostic methods used. The students received a detailed briefing from the doctors, initially carried out the assigned tasks under supervision, and were allowed to work independently if they performed satisfactorily.

### 2.3. Instruments

All pupils underwent a detailed cardiovascular examination, including a questionnaire comprising items on their health status, physical activity, and sedentary behavior. The survey was created with REDCap 14.6.7. Comprehensive data were collected on health and fitness: frequency of daily and weekly physical activity, school grade in Physical Education, swimming ability, steps during the day, sedentary behavior, and screen time during the week and on weekends. The questionnaires were answered under pseudonyms without the presence of parents and teachers to ensure the honesty of the children.

Firstly, the participants were asked whether they would describe themselves as fit. This question could be answered with yes or no. With regard to physical activity, the students were first asked how long they were physically active per day in a typical week; the activities were categorized into moderate to vigorous intensity, vigorous intensity, and those that reinforce muscle strength, in accordance with the WHO guidelines [22]. The answer options were less than an hour, one hour, one to two hours, or more than two hours for moderate to vigorous activity and less than once a week, once a week, two to three times a week, and more than three times a week for vigorous activity and those that strengthen the muscles. According to the WHO guidelines, at least 60 min of moderate to vigorous activity per day as well as performing vigorous activity and those strengthening the muscles at least three times a week were defined as being in line with the recommendations. The pupils were also asked if they were members of a sports club. If the answer was yes, the type of sport should be indicated. In the questionnaire, the students were also asked to indicate the sports grade of their last school report: the possible answers were 1 to 6, with 1 being the best and 6 the worst grade in the German school system. They were also asked whether they could swim, with the answer options ranging from yes very well to a little to no. The students were also asked whether they could swim 100 m at a stretch. The students who measured their daily step count with their mobile phone should indicate whether they take less than 5000 or less or more than 10,000 steps a day. If daily step count was not measured, the answer option “do not track” was to be chosen. Screen time was to be given in full hours, once for weekdays and once for weekends, with possible answers ranging from 1 to 8 h. In addition, the daily sitting time was surveyed; here, the participants could choose between less than three, 3–5 h, 6–7 h, or more than 7 h. Finally, the students were asked whether they have sleep problems and how many hours they sleep per night during the week or at the weekend (<5, 5–6, 6–7, 8–9, >10 h). The questionnaires were analyzed by a team of three people according to the four-eyes principle using a fixed scheme.

The weight and height of the participants was measured on-site by our study team. The participants were asked to remove shoes, heavy clothing, jewelry, and any items in their pockets. The weight was assessed with portable electronic scales. The children were asked to stand in the center of the scales and let their arms hang loosely beside their bodies. They were instructed to stand still to enable an accurate measurement. The weight was recorded in kg to one decimal place when the scale showed a fixed value on the display. For height measurement, the participants were asked to stand up straight against a wall, making sure that their shoulders were at the same height, their knees were together, and their arms were hanging beside their bodies. The head, buttocks, or heels should touch the wall in at least two places. The children were asked to stand still and fixate on a point straight in front of them. The height was measured using a board which was placed on a right angle to the measuring stick. The values were measured to the nearest half a centimeter and rounded down if necessary.

The following formula was used to calculate the body mass index (BMI). Overweight and obesity were defined as recommended by the German obesity society (overweight weight percentile > 90–97, obesity if weight percentile > 97) [23].

Physical fitness was measured using a stair-climbing test, a standardized exercise test that was further developed and adapted by the Department of Pediatric Cardiology at the LMU [24]. The test was carried out on-site by the study team and subsequently evaluated. The test consists of running up and down four floors as quickly as possible, where each step must be taken individually. All participants were cheered on with the same phrases. The time required was measured in seconds using a stopwatch. Age- and gender-dependent cut-off values were used for the test. Based on the time achieved, the fitness of the participants was rated as good (males <10 y 40–55s, males >10 y 40–50s, females all ages 40–55s), moderate (males <10 y 55–75s, males >10 y 50–65s, females <10 y 55–75s, females >10 y 55–70s), or poor (males <10 y >75s, males >10 y >65s, females <10 y >75s, females >10 y >70s).

Physical activity was defined as the number of hours spent engaging in sport according to the questionnaire completed by the students, while physical fitness was the result of the stair-climbing test, which was carried out on-site.

### 2.4. Statistical Analysis

The characteristics of the study population, such as sex, age, BMI, and school level, were compared between the categories of screen time (within recommendations, ≤2 h versus exceeding recommendations, >2 h) during the weekday and weekend [19]. The key outcome variables, including sports grade, self-assessed fitness, swimming ability, and PA behaviors (steps per day, time spent on PA per day, and time spent on high-pulse and muscular PA per week), were summarized and compared between males and females. To examine group differences for continuous variables, Student’s *t*-tests were performed, and to evaluate differences between proportions of categorical variables, chi-square tests were used. Univariate logistic regression models were employed to explore the relationship between sex and binary outcomes and to quantify changes in mean BMI by sport club membership. Multivariable logistic models were adjusted for age. Effect sizes were reported where relevant (odds ratios with 95% confidence intervals). Statistical significance was set at an alpha level of 0.05. All analyses were conducted using R version 4.4.2 (2024-10-31 ucrt) [25].

## 3. Results

Of the total number of pupils enrolled in this ongoing study (*n* = 922), 883 pupils aged between 8 and 18 were included in the analysis. The characteristics are shown in Table 1. The mean (SD) age of the children was 13.1 (2.4) years, and the proportion of girls was 46% (*n* = 406). The mean (SD) BMI of the included pupils was 19.4 (3.7) kg/m^2^. Most of the children and adolescents (68.4%) attended secondary school.

In terms of media consumption, 324 children spent 2 h or less in front of a screen during the week, whereas 559 children spent 3 h or more. On the weekend, nearly 3 in 4 children (670/883) indicated >2 h of screen time, while 24.1% (213/883) indicated ≤2 h.

Regarding differences in screen time, no significant differences were found between males and females, regardless of day of the week. However, compared to children with ≤2 h of screen time during the week, children who had screen time >2 h during the week were older (mean [SD]: 13.9 [2.1] years versus 11.7 [2.2]), had a higher BMI (mean [SD]: 20.2 [3.7] kg/m^2^ versus 18.0 [3.2] kg/m^2^), and were more frequently in secondary school (74.2% versus 58.3%). The results were similar for screen time on weekends.

### 3.1. Influence of Screen Time on Sport Outcomes

As shown in Table 2, children who had >2 h of screen time (versus ≤2 h) during the week were 3.23 (95% CI 1.76, 5.95) times more likely to receive a grade of 3 or worse in sports and 2.35 (95% CI 1.68, 3.29) times more likely not to be a member of a sports club. The association remained significant after adjusting for age. We found similar results for screen time on weekends, although the association between sports club membership and weekend screen time disappeared after adjusting for age.

### 3.2. Adherence on Screen Time and Physical Activity Recommendations

**Figure 1:**
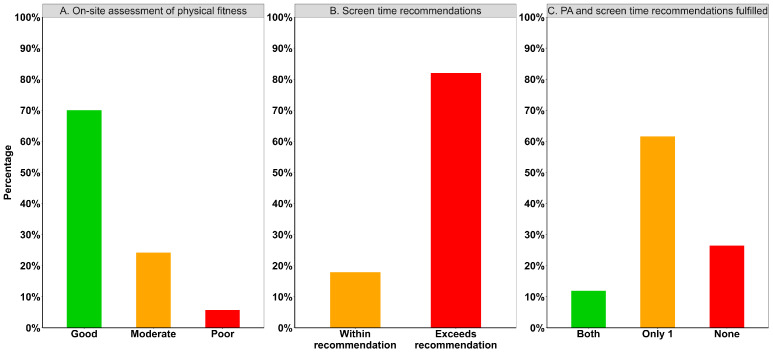
Proportion of children adhering to screen time and physical activity recommendations. (**A**) shows the results of the physical fitness assessment on-site. (**B**) shows proportion of pupils within or exceeding screen time recommendations. (**C**) shows proportion of children who fulfill both, only 1, or none of the recommendations.

The evaluation of the pupils’ physical fitness revealed that 30% showed moderate to poor fitness and 70% showed a good fitness level (Figure 1A). Regarding the recommendations for screen time, only about 17.9% of the children met the recommendations (Figure 1B). Considering both physical activity and screen time together, it was found that only about 11.9% of the study participants met the recommendations for both, about 61.6% met one of the two, and 26.5% did not follow either recommendation (Figure 1C).

### 3.3. Lifestyle Indicators and Adherence to Screen Time and Physical Activity Recommendations

**Figure 2:**
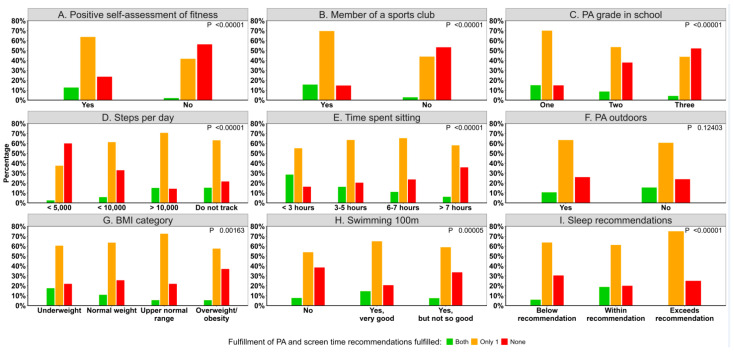
Lifestyle indicators according to adherence to screen time and physical activity recommendations. *p*-values are based on χ2 test.

Lifestyle indicators and adherence to physical activity and screen time recommendations are depicted in Figure 2. Approximately 56.3% of children who self-reported that they were not fit did not meet either the recommendations on screen time nor the ones for physical activity (Figure 2A). A large proportion of the children who did not fulfil both of the two recommendations for physical activity and screen time were not members of a sports club (53.3%, Figure 2B), had a school grade of ≥3 in Physical Education (52.1%, Figure 2C), walked <5000 steps per day (60%, Figure 2D), spent ≥7 h sitting (35.8%, Figure 2E), had a high proportion of overweight or obese (40%, Figure 2G), were non-swimmers (38.5%, Figure 2H), and slept for less than the recommended hours (30%, Figure 2I).

## 4. Discussion

The prevention project hand-on-heart (“Hand-aufs-Herz”) is an ongoing project that has collected data from almost 900 German school children. The children and adolescents completed a questionnaire which included questions on media consumption, daily step count, membership in a sports club, and a self-assessment of their daily physical activity. Biometric data such as height, weight, and BMI were measured on-site. This study aimed to investigate the influence of screen time on children’s lifestyle and physical fitness.

The results of our study raise concerns. Only around 20% of children complied with the recommended limit of, depending on age, 1–2 h of screen time per day. If the daily recommended minimum duration of physical activity is also considered, the proportion of children who followed both recommendations fell to only 10%. In a comparable study, higher adherence to the recommendations was found, but the results were still worrying: only around a third of the 15,000 included children met the recommendations for screen time and physical activity [20]. The recommendations are based on the guidelines of the Federal Centre for Health Education (BzgA) and the WHO and are supported by several German medical associations like the German Society for Paediatric Cardiology [19,22,26].

Children today grow up with the daily use of media [14]. Our study found that almost 3 out of 4 children spend 3 h or more in front of a screen on the weekend. Other studies confirmed these results. In more than 100,000 children aged 6 to 14, a similar average screen time of almost 3 h per day was found, a value that is considered too high at any age according to the guidelines [19,27]. These studies clearly demonstrate that high media consumption is prevalent. It should be kept in mind that excessive use can have negative consequences on health and behavior. Obesity can be a consequence of media consumption and is encouraged by it [28]. Excessive media use can also lead to sleep disorders, poorer school performance, and behavioral problems [29].

In the present study, the consequences of excessive digital media use were identified: children spending more hours in front of a screen are more likely to be physically inactive. These results were confirmed by another study, which linked screen time to physical activity in everyday life [30]. In addition, this study found that children’s BMI was positively correlated with screen time. Children who spent less than two hours in front of a screen at weekends had an average BMI of 17.6, while children with more than two hours of screen time had an average BMI of 20.

This was further validated by a study that also showed a correlation between BMI, the amount of digital media consumption, and physical activity. Children who were overweight tended to exercise less and spend more time in front of screens. The screen time of children with a BMI of <90. Pzt. was 12.6 ± 9.7 h/week vs. the screen time of those with a BMI of >90. Pzt., which was 18.3 ± 15.7 h/week. A total of 57% of normal-weight children also took part in sport outside of school, compared to only 32% of overweight children [31].

It was additionally observed that non-compliance with the recommendations for physical activity and screen time can have an impact on lifestyle. A large proportion of the children who did not meet either recommendation did not attend a sports club, walked less than 5000 steps per day, sat for more than 7 h per day, scored 3 or worse in sports, and were overweight or obese. Research has also previously demonstrated that there is a correlation between exercise behavior, dietary habits, and smoking status [3]. Digital media consumption increases with age [18]. The results of this study also show that older children were more likely to have screen time that exceeded the recommendations.

In summary, it can be said that this study produced similar results to preceding studies. Children with too much screen time also displayed sedentary behavior and had a higher risk of becoming overweight. They were also less likely to be members of a sports club and had a lower daily step count. The special feature of this study is that, in addition to the self-assessment, physical fitness was analyzed on-site using a stair-climbing test [24].

Exercise has many positive effects on the body and the mind. However, less active lifestyles have increased the rate of obesity and associated health problems [32]. Physical activity has important health benefits, including lower blood pressure, lower BMI, and improved triglyceride levels [3]. Fitness is an independent risk factor for cardiovascular disease and should be given special consideration [3]. The fitness of children and adolescents decreases with increasing media consumption, which underlines the need to take preventive measures at an early stage. At the same time, it is evident that the younger generation’s interest in digital media is growing steadily, and their daily use of it has become indispensable in everyday life.

Considering the results of this study, it is crucial to develop preventive measures and continue to implement existing ones. Parents are cautioned and asked to consider media use according to the age, health, and developmental stage of each child [14]. Further studies show how important it is to develop a health promotion program that includes promoting the recommended amount of physical activity and screen time [20,33]. In addition, the focus should be directed towards more physical activity for children and adolescents in schools [3].

With regard to the problem of overweight children, it was found that simply increasing physical activity only brings about limited benefits. Rather, it was shown that reducing sedentary behavior is a much more effective strategy for preventing and reducing obesity [3]. In contrast, a meta-analysis showed that although interventions on media consumption could reduce children’s screen time, no effect on BMI was achieved [34]. For our study, this means that although a link between high media consumption, obesity, and lack of exercise was found, the solution does not lie in reducing screen time alone. Rather, lifestyle, especially sedentary behavior, should also be considered. In this regard, holistic lifestyle interventions and their impact on obesity in children and adolescents should be further studied, preferably with larger sample sizes [34].

It also became clear that the real-life effects of measures to promote physical activity can only be determined if sufficient previous comparative data are available [30]. In order to obtain better data, fitness should be measured in an objective manner, and the result should then be compared to the children’s statements. Therefore, we included a dedicated and standardized fitness test in our screening process. As fitness is an independent risk factor for cardiovascular disease, the focus should not only be on surveying the media consumption of children and adolescents but be, in particular, on the objective and meaningful recording of fitness in order to develop targeted preventive measures on this basis.

Despite efforts to create a standardized setting, our study has some limitations.

Daily and weekly physical activity and screen time were collected using a questionnaire that required a certain sense of time. This ability may not yet be fully developed in younger children. Questionnaires were also used in other studies to assess these aspects, although age dependency could play a role, such as in a study with 9- to 18-year-olds [20].

In addition to a basic sense of time and understanding, the value of the data collected was based on the honesty of the answers collected. However, children may have concealed their actual habits for fear of consequences. One study tried to circumvent this by having parents fill out the questionnaires, but this could lead to bias as they often do not know their children’s media consumption and activity in detail [31].

In addition to the steps already taken, as outlined in the Methods Section, better documentation of screen time without being dependent on the subjective assessment of the children could be made possible by recording the time using the electronic devices themselves, which already record screen time.

## 5. Conclusions

Our results confirm the negative effects of too much screen time on the lifestyle of children and adolescents, which have already been investigated. We show that pupils showing excessive media consumption are more likely to consider themselves as not fit and conducted a standardized fitness test to objectify this finding.

## Figures and Tables

**Table 1:** Characteristics of the hand-on-heart-study population.

Characteristics	All Children	Screen Time During Weekday			Screen Time During Weekend		
		≤2 h	>2 h	*p*-Value	≤2 h	>2 h	*p*-Value
Number of pupils, N	883	324	559		213	670	
Sex				0.292			0.805
Male	477 (54.0)	167 (51.5)	310 (55.5)		113 (53.1)	364 (54.3)	
Female	406 (46.0)	157 (48.5)	249 (44.5)		100 (46.9)	306 (45.7)	
Age, years	13.1 (2.4)	11.7 (2.2)	13.9 (2.1)	<0.001	11.3 (2.2)	13.6 (2.1)	<0.001
Body mass index (BMI), kg/m^2^	19.4 (3.7)	18.0 (3.2)	20.2 (3.7)	<0.001	17.6 (3.1)	20.0 (3.7)	<0.001
School level				<0.001			<0.001
Primary school	100 (11.3)	81 (25.0)	19 (3.4)		63 (29.6)	37 (5.5)	
Middle school	58 (6.6)	10 (3.1)	48 (8.6)		9 (4.2)	49 (7.3)	
Secondary school	604 (68.4)	189 (58.3)	415 (74.2)		111 (52.1)	493 (73.6)	
High school	121 (13.7)	44 (13.6)	77 (13.8)		30 (14.1)	91 (13.6)	

Data are mean (SD) or *n* (%) and tested with regard to screen time per weekday or weekend using Student’s *t*-test for continuous and χ2 test for categorical variables.

**Table 2:** Sports grade and sports club membership by screen time.

Outcome		Screen Time During Weekday		Screen Time During Weekend	
	N	Unadjusted	Age-Adjusted	Unadjusted	Age-Adjusted
Sports grade at school					
1	524	Reference	Reference	Reference	Reference
2	288	**2.14 (1.57, 2.92)**	**1.80 (1.27, 2.54)**	**1.93 (1.35, 2.76)**	**1.52 (1.03, 2.25)**
≥3	71	**3.23 (1.76, 5.95)**	**2.82 (1.46, 5.42)**	**3.28 (1.53, 7.00)**	**2.69 (1.20, 6.01)**
Sports club membership					
Yes	639	Reference	Reference	Reference	Reference
No	244	**2.35 (1.68, 3.29)**	**1.75 (1.22, 2.53)**	**2.13 (1.44, 3.14)**	1.48 (0.97, 2.25)

Values are odds ratios and 95% confidence intervals based on logistic regression. The influence of screen time ≥ 3 (test) versus ≤2 h (control) was assessed for each outcome. Bold font indicates *p* < 0.05.

## Data Availability

Data cannot be shared publicly because the participants did not provide explicit consent for data sharing in accordance with the European Union’s General Data Protection Regulation and relevant German privacy laws. However, data can be made available to researchers who meet the criteria for access to confidential information through the Research Ethics Board of Ludwig-Maximilians-Universität Munich, Germany. Requests should be directed to ethikkommission@med.uni-muenchen.de.

## Abbreviations

The following abbreviations are used in this manuscript:

BMI	Body mass index
ORs	Odds ratio
PA	Physical activity
SD	Standard deviation
WHO	World health organization
